# Frailty Among Breast Cancer Survivors: Evidence From Swedish Population Data

**DOI:** 10.1093/aje/kwad048

**Published:** 2023-03-07

**Authors:** Alexandra M Wennberg, Anthony Matthews, Mats Talbäck, Marcus Ebeling, Stina Ek, Maria Feychting, Karin Modig

**Keywords:** aging, breast cancer, cohort study, frailty

## Abstract

Incidence and survival of breast cancer, the most common cancer among women, have been increasing, leaving survivors at risk of aging-related health conditions. In this matched cohort study, we examined frailty risk with the Hospital Frailty Risk Score among breast cancer survivors (*n* = 34,900) and age-matched comparison subjects (*n* = 290,063). Women born in 1935–1975, registered in the Swedish Total Population Register (1991–2015), were eligible for inclusion. Survivors had a first breast cancer diagnosis in 1991–2005 and survived ≥5 years after initial diagnosis. Death date was determined by linkage to the National Cause of Death Registry (through 2015). Cancer survivorship was weakly associated with frailty (subdistribution hazard ratio (SHR) = 1.04, 95% confidence interval (CI): 1.00, 1.07). In age-stratified models, those diagnosed at younger ages (<50 years) had higher risk of frailty (SHR = 1.12, 95% CI: 1.00, 1.24) than those diagnosed at ages 50–65 (SHR = 1.03, 95% CI: 0.98, 1.07) or >65 (SHR = 1.09, 95% CI: 1.02, 1.17) years. Additionally, there was increased risk of frailty for diagnoses in 2000 or later (SHR = 1.15, 95% CI: 1.09, 1.21) compared with before 2000 (SHR = 0.97, 95% CI: 0.93, 1.17). This supports work from smaller samples showing that breast cancer survivors have increased frailty risk, particularly when diagnosed at younger ages.

## Abbreviations

CI: confidence interval
HFRS: Hospital Frailty Risk Score
IR: incidence rate
IRR: incidence rate ratio
SHR: subdistribution hazard ratio

Breast cancer is the most common form of cancer among women, and while mortality has declined substantially in the past 4 decades, incidence has increased (1, 2). However, the combination of a decrease in cancer mortality, increase in incidence, and increasing older adult population means that the number of aging cancer survivors is growing. While that is largely positive, it does present new challenges. Many cancer survivors have a higher risk of death than the general population as they age (3), and it is possible that cancer survivors are also at higher risk of aging-related conditions, including frailty. As of 2020, more than two-thirds of all cancer survivors are aged 65 years or older (4). Although most longitudinal studies on cancer survivorship have been conducted in childhood cancer survivors, incidence of cancer increases dramatically with increasing age, so it is critical to examine aging trends in survivors of both childhood and adult cancer. Older survivors are more likely to have more chronic health problems, and it is imperative that we understand the health challenges of aging survivors to provide the long-term care and interventions needed to minimize these morbidities and their effects. Indeed, long-term care guidelines for aging cancer survivors, which can be implemented in primary care settings, are needed (5).

Compared with those without a history of cancer, observational evidence has suggested that cancer survivors are at increased risk of cognitive decline, frailty, functional decline, and chronic age-related conditions, even before the age of 65 (6, 7). Frailty, a chronic condition characterized by vulnerability and a weakened state (8), can be defined in 2 ways: phenotypically, representing an underlying dysregulation in energy, or as an accumulation of deficits, determined by summing an individual’s conditions and impairments. Frailty affects up to 25% of community-dwelling older adults over age 65 years and increases with age (9); it is one of the leading conditions associated with poor quality of life and mortality (8). The limited available evidence from cross-sectional studies in smaller clinical samples of one type of cancer show that rates of frailty in adult cancer survivors are double or even quadruple the rates seen in community-dwelling counterparts (10–12). One early study among a small sample of breast cancer survivors showed that they had higher prevalence of frailty compared with other community-dwelling women (13). It seems that not only are cancer survivors at higher risk of developing these outcomes, but they are also at higher risk at younger ages (6, 7). However, examining this in larger, population-based samples is critical for understanding larger trends and risk factors.

Overall, prevalence and risk of frailty among survivors of adult cancers is not well-defined. Understanding the long-term health consequences in aging cancer survivors is critical to ensuring high quality of life among this growing population (5, 14). Further, measures of poorer quality of life have been associated with mortality in cancer patients, giving further importance to understanding aging well in cancer survivors (15, 16). To that end, the aim of this study was to investigate the prevalence of frailty and the risk of developing frailty in breast cancer survivors compared with women without history of cancer.

## METHODS

### Design and participants

This study was designed as a matched cohort study where we examined risk of developing frailty among adult breast cancer survivors. Women born in 1935–1975 who were registered in the Swedish Total Population Register (TPR) between January 1, 1991, and December 31, 2015, were eligible to be in the study. Subjects were followed from their first record in TPR or their last immigration before their first TPR record (1991 or later) until their first emigration after their last TPR record, death, or end of follow-up. Breast cancer survivors were defined as women with a first breast cancer diagnosis registered in the National Cancer Register between 1991 and 2005, who survived for 5 years after their initial diagnosis. There were 44,780 women with a first breast cancer diagnosis between 1991 and 2005; 6,029 of these women died or were censored within 5 years of the diagnosis and were thus not included in the final analyses (Web Figure 1, available at https://doi.org/10.1093/aje/kwad048). Comparison subjects were randomly selected from the cohort members who were at risk at the time of the survivors’ diagnoses of breast cancer. Comparisons were initially defined as women with no diagnosis of breast cancer (or who had not yet received a breast cancer diagnosis at the date of matching). We then additionally excluded women with history of any other type of cancer from the analyses. Initially, we identified 447,800 comparison subjects matched to survivors on age at diagnosis. Initially, comparisons were matched to survivors on a 10:1 basis; however, in the final sample, all survivors had at least 6 comparisons, and 30,010 survivors had 10 comparisons after censorship. Of the comparison subjects, 60,290 were censored within the first 5 years, along with their matched cases, and 9,975 died or were censored (e.g., emigrated, developed breast cancer) in the first 5 years after case diagnosis. The final cohort for analysis included 38,751 survivors and 377,535 comparison subjects, 4.6% of whom developed breast cancer during follow-up and were thus censored at that point.

We measured frailty using the Hospital Frailty Risk Score (HFRS), a cumulative deficit measure that uses 109 *International Classification of Diseases*, *Tenth Revision*, hospital codes that are weighted (0.1 to 7.1) and summed to create a frailty score (17). The HFRS was designed as a screening tool for frailty using routinely collected clinical data; it has been validated in multiple populations (18, 19) and overlaps with the gold-standard Rockwood frailty index (17). Hospitalizations and specialist outpatient care were identified through linkage of the cohort with the National Patient Register (20), which has coded diagnoses according to *International Classification of Diseases*, *Tenth Revision*, since 1997. We additionally translated the codes to be used with the *International Classification of Diseases*, *Ninth Revision*, as well, to allow for longer follow-up (Web Table 1). Inpatient records were used from 1995–2015 and outpatient from 2001–2015; duplicate codes at the 3-character level were removed. Frailty was assessed using the diagnostic codes from all hospitalizations and outpatient care visits during an entire year for each subject; thus frailty was ascertained on a yearly basis, consistent with previous work (21). If an individual did not seek inpatient or outpatient care for a given year, they were assigned a score of 0 on the HFRS. Individuals were considered frail once their frailty score exceeded 5 (17). Death and date of death were determined by record linkage to the National Cause of Death Registry through December 31, 2015 (end of follow-up) (22).

### Statistical analysis

Participant baseline characteristics are presented in Table 1. We additionally examined HFRS and prevalence of frailty 1 year prior to breast cancer diagnosis, to account for the possibility that women who develop breast cancer may be different in terms of health status prior to cancer diagnosis. Incidence rates (IRs) for frailty were calculated by taking the number of frail cases divided by the person-time at risk. Crude incidence rate ratio (IRR) was derived by dividing the frailty incidence rate for breast cancer survivors by the rate for comparison subjects. We examined rates overall and by age categories (i.e., 30–39, 40–49, 50–59, 60–69) and period (i.e., before and after 2000) of diagnosis.

**Table 1:** Baseline and Prior Characteristics of Breast Cancer Survivors and Comparison Subjects in Swedish National Register Data, 1991–2015

	**All (*n* = 324,963)**			**Survivors (*n* = 34,900)**			**Comparisons (*n* = 290,063)**		
**Participant Characteristic**	**No.**	**%**	**Mean (SD)**	**No.**	**%**	**Mean (SD)**	**No.**	**%**	**Mean (SD)**
Age at diagnosis, years			57.3 (8.2)			57.6 (8.0)			57.3 (8.2)
Country/region of birth									
Sweden	279,746	86.1		30,818	88.3		248,928	85.8	
Other Nordic country	19,059	5.9		1991	5.7		17,068	5.9	
Europe	15,079	4.6		1,398	4.0		15,079	5.2	
Outside of Europe	9,681	3.0		693	2.0		8,988	3.1	
HFRS score 5 years after diagnosis			1.51 (2.49)			1.62 (2.55)			1.49 (2.48)
HFRS at diagnosis			0.06 (0.47)			0.08 (0.50)			0.06 (0.50)
HFRS 1 year prior to diagnosis			0.04 (0.39)			0.04 (0.35)			0.02 (0.27)
<50 years of age at diagnosis			0.02 (0.27)			0.02 (0.28)			0.02 (0.27)
50–65 years of age at diagnosis			0.04 (0.34)			0.03 (0.34)			0.04 (0.35)
>65 years of age at diagnosis			0.08 (0.55)			0.06 (0.43)			0.08 (0.56)
Frailty 1 year prior to diagnosis	438	0.12		38	0.10		400	0.12	
Cumulative frailty in 5 years from diagnosis to start of follow-up	5,399	1.5		554	1.5		4,845	1.5	
Follow-up time, years			9.3 (4.6)			8.8 (4.8)			9.3 (4.6)

Abbreviations: HFRS, Hospital Frailty Risk Score; SD, standard deviation.

We then used subdistribution hazard ratio models to examine risk of frailty over time, specifying death as a competing risk. Competing risk models are a type of survival analysis that accounts for a competing event (e.g., death) that is likely to occur to the participants during follow-up. Unlike traditional survival analysis methods, these models are less likely to overestimate the risk of the outcome. Competing risks are events that hamper the observation of the event of interest or modify the chance that the event of interest occurs. We chose to use competing risk models, because although mortality risk in cancer patients has decreased in recent years, they are still at higher risk of death compared with individuals without cancer (1). Model 1 adjusted for age at diagnosis, and model 2 adjusted for age at diagnosis and country/region of birth, because evidence suggests that race/ethnicity may have an impact on the association between cancer survivorship and health outcomes (23). Subsequently, we stratified the analyses by age at diagnosis (<50, 50–65, >65), because of the wide range of ages included in the sample. Finally, because until the late 1990s chemotherapy agents caused substantial apoptosis of normal cells (24), we additionally stratified the analysis by year of cancer diagnosis (before 2000 vs. 2000 or later). We then additionally used the traditional Cox proportional hazards model, censoring death to compare the hazard ratios with the subdistribution hazard ratios. All statistical analyses were conducted using Stata, version 16 (StataCorp, LLC, College Station, Texas).

## RESULTS

The mean age at breast cancer diagnosis was 57 years (Table 1). The mean HFRS at the year of diagnosis was 0.08 (standard deviation, 0.50) for breast cancer survivors and 0.06 (standard deviation, 0.50) for comparison subjects. One year prior to diagnosis, the mean HFRS was 0.04 for both survivors and comparison subjects, and approximately 1 per 1,000 subjects in each group were frail. In the cumulative 5 years between diagnosis and beginning of follow-up, 1.5% of both survivors and comparison subjects became frail.

**Figure 1:**
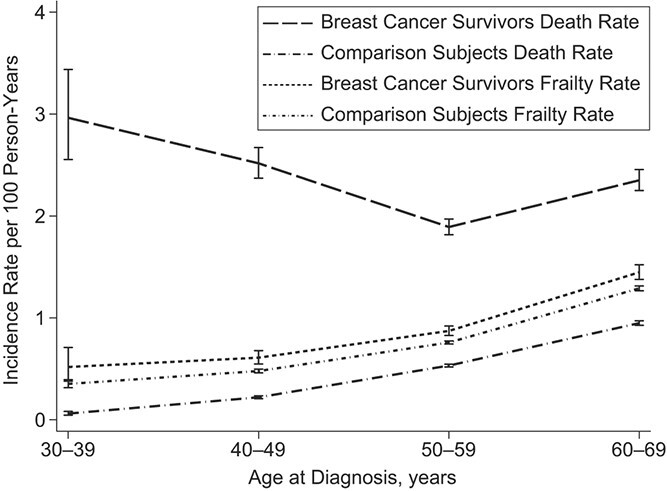
Incidence (per 100 person-years) of frailty and mortality for breast cancer survivors and comparison subjects, Sweden, 1991–2015. Breast cancer survivors and comparison subjects, stratified by age at breast cancer diagnosis, showed increasing frailty and mortality with age but convergence trends between breast cancer survivors and comparison subjects.

Five years after diagnosis, the overall incidence of frailty was higher in breast cancer survivors (IR = 10.52, 95% confidence interval (CI): 10.18, 10.87) compared with comparison subjects (IR = 8.99, 95% CI: 8.89, 9.10). The relative difference was 17% higher incidence (IRR = 1.17, 95% CI: 1.13, 1.21) (Table 2). Stratified by age of diagnosis, we observed the largest IRR among those aged 30–39 at diagnosis (1.47, 95% CI: 1.03, 2.06). The difference in incidence rate decreased with increasing age of diagnosis but remained consistently higher among breast cancer survivors compared with comparison subjects. We additionally saw that among those diagnosed in 2000 or later, there was a greater difference between breast cancer survivors and comparison subjects (IRR = 1.24, 95% CI: 1.17, 1.31) than the difference between those diagnosed before 2000 and their comparison subjects (IRR = 1.13, 95% CI: 1.08, 1.18). Among those aged 30–39 at diagnosis, the difference in frailty between breast cancer survivors and comparisons was much smaller than the difference in mortality (Figure 1). These differences shrank with age, so among those aged 40–49 years, the incidence rate of frailty among survivors was greater, as was incidence rate of death. This trend then continued among those aged 50–59 at diagnosis, where incidence rate of frailty among survivors was higher but closer to that among cancer free women, with a similar trend in incidence rate of death among survivors and comparisons. However, there was a very slight widening of this frailty incidence rate gap among those aged 60–69 at diagnosis, with a higher incidence rate of death.

**Table 2:** Frailty Incidence Rates (per 1,000 Person-Years) by Age or Period of Diagnosis for Breast Cancer Survivors and Comparison Subjects, and Incidence Rate Ratios, in Swedish National Register Data, 1991–2015

	**Survivors**				**Comparison Subjects**				**IRR**	**95% CI**
**Grouping**	**Frail**	**PY**	**IR**	**95% CI**	**Frail**	**PY**	**IR**	**95% CI**		
Overall	3,541	336,563.3	10.52	10.18, 10.87	26,629	296,0746.1	8.99	8.89, 9.10	1.17	1.13, 1.21
By age of diagnosis										
30–39	39	7,518.3	5.19	3.79, 7.10	306	86,808.4	3.53	3.15, 3.94	1.47	1.03, 2.06
40–49	330	54,088.4	6.10	5.48, 6.80	2,608	544,063.5	4.79	4.61, 4.98	1.27	1.13, 1.43
50–59	1,362	155,894.8	8.74	8.28, 9.21	10,113	1,331,764.9	7.59	7.45, 7.74	1.15	1.09, 1.22
60–69	1,566	108,186.4	14.48	13.78, 15.21	11,646	901,893.5	12.91	12.68, 13.15	1.12	1.06, 1.18
By period of diagnosis										
Before 2000	2,095	212,494.6	9.86	9.45, 10.30	15,576	1,783,863.3	8.73	8.60, 8.87	1.13	1.08, 1.18
2000 or later	1,446	124,068.8	11.65	11.07, 12.27	11,053	1,176,882.8	9.39	9.22, 9.57	1.24	1.17, 1.31

Abbreviations: CI, confidence interval; IR, incidence rate; IRR, incidence rate ratio; PY, person-years.

**Figure 2:**
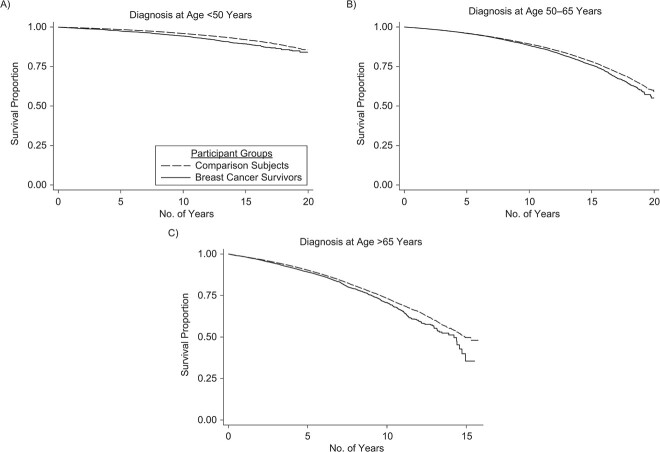
Competing risks analysis survival curves, stratified by age in years at breast cancer diagnosis, Sweden, 1991–2015. Breast cancer survivors diagnosed at ages <50 (A), 50–65 (B), and >65 (C) years are at greater risk of frailty over follow-up.

In the age-adjusted subdistribution hazard model taking competing risk of death into account, we found breast cancer survivors, compared with those without history of cancer, had a small but elevated risk of developing frailty (subdistribution hazard ratio (SHR) = 1.04, 95% CI: 1.00, 1.07) (Table 3). Adjusting for age at diagnosis and country/region of birth had no effect on the estimates. However, in age-stratified analyses we found that those diagnosed before the age of 50 had a higher risk of frailty (SHR = 1.12, 95%: 1.00, 1.24) compared with those diagnosed at ages 50–65 (SHR = 1.03, 95% CI: 0.98, 1.07) and those aged over 65 at diagnosis (SHR = 1.09, 95% CI: 1.02, 1.17). Survival curves show that breast cancer survivors were more likely to become frail over time, in all diagnosis age groups, even when accounting for competing risks of death (Figure 2). In analyses stratified by year of diagnosis (before 2000 vs. 2000 or later) and adjusted for age at diagnosis and country/region of birth, those diagnosed with breast cancer later had a higher risk of frailty (SHR = 1.15, 95% CI: 1.09, 1.21) than those diagnosed earlier (SHR = 0.97, 95% CI: 0.93, 1.17). Results from standard Cox proportional hazards models showed similar, although slightly stronger, associations between survivorship status and risk of frailty (Web Table 2).

**Table 3:** Subdistribution Hazard Ratios Comparing Breast Cancer Survivors to Comparison Subjects in Swedish National Register Data, 1991–2015

**Model**	**Total**	**No. of Failures^a^**	**No. of Competing^a^**	**SHR**	**95% CI**
Model 1^b^	324,963	30,170	20,474	1.04	1.00, 1.07
Model 2^c^				1.04	1.00, 1.08
Stratified by age					
<50 years of age at diagnosis	60,888	3,285	2,280	1.12	1.00, 1.24
50–65 years of age at diagnosis	203,099	19,221	13,312	1.03	0.98, 1.07
>65 years of age at diagnosis	60,976	7,664	4,882	1.09	1.02, 1.17
Stratified by period of diagnosis					
Diagnosed before 2000	149,234	17,671	13,137	0.97	0.93, 1.17
Diagnosed 2000 or after	175,729	12,499	7,337	1.15	1.09, 1.21

Abbreviations: CI, confidence interval; SHR, subdistribution hazard ratio.

^a^ Failures refers to the number of people who developed frailty, and competing refers to the number of people who died.

^b^ Model 1 adjusted for age.

^c^ Model 2 adjusted for age and country/region of birth.

In sensitivity analyses, we repeated the analyses including comparison of subjects with a history of another type of cancer besides breast cancer. In these analyses, the association between breast cancer survivorship and frailty was less robust (SHR = 1.02, 95% CI: 0.99, 1.06). Further, we excluded those who had an HFRS score of >5 the year prior to cancer diagnosis and found that the association did not change (SHR = 1.04, 95% CI: 1.00, 1.07). We additionally adjusted for HFRS score 1 year prior to diagnosis and again did not find any differences in the results (SHR = 1.04, 95% CI: 1.00, 1.07). Moreover, we examined risk of frailty from 1 year after diagnosis, instead of 5 years, and found that the association trended towards the null (SHR = 0.93, 95% CI: 0.88, 0.98), although many individuals who had been diagnosed with cancer died within this period. We then stratified by follow-up time to understand whether the differences in age and year at diagnosis were attributable to differences in follow-up time but found that this did not explain the differences (results not shown). In post hoc analyses, we examined which diagnoses were most common in the breast cancer survivor and comparison subject groups, to determine which conditions were driving the HFRS. We found that unspecified conditions were most common (e.g., other and unspecified osteoarthritis, other joint disorders, falls, urinary disorders, and other soft tissue disorders, not elsewhere classified).

## DISCUSSION

In this study, we examined the risk of developing frailty among breast cancer survivors compared with age-matched women without a previous cancer diagnosis. Five years past their initial diagnosis, breast cancer survivors had a small but elevated risk of developing frailty at any age compared with women who had no history of cancer at the time of diagnosis of the matched breast cancer patient. However, the association was seen primarily among breast cancer survivors diagnosed at earlier ages. The risk of death was high among breast cancer survivors, particularly in sensitivity analyses that included the first 5 years immediately after diagnosis, which is consistent with our earlier findings (25). Further, when stratifying by calendar period of diagnosis, we found an association only among those diagnosed with breast cancer in 2000 or later, while there was no association in those diagnosed prior to 2000. Given that therapeutics became less toxic after the late 1990s, a reduced risk of frailty could have been expected (24). On the other hand, 5-year survival has increased over time, reducing the competing risk of death, which means that more patients might make it beyond 5 years, but potentially with an increased risk of frailty. One factor affecting improved 5-year survival is changes in breast cancer screening guidelines in Sweden in the late 1990s, which likely led to earlier detection (1).

Our findings echo those from other studies. Frailty in adult cancer survivors ranges from 9% to nearly 60%—double to quadruple the rates seen in age- and sex-matched populations (26). A study in breast cancer survivors aged 65 or older showed that they are at increased risk of frailty compared with aged-matched controls without a history of cancer, with the strongest association among younger (ages 65–70) survivors (13). Notably, these studies used both cumulative deficit measures of frailty, similar to what we used, and phenotypic measures (e.g., Fried frailty score). Together with these findings, our results indicate that, whether a cumulative deficit or phenotypic framework is used as the basis of frailty measurement, cancer survivors show increased risk of developing frailty. Although cumulative deficit models of frailty measurement overlap with measures of multimorbidity, tools such as the HFRS have been validated as a measure of frailty, showing overlap with gold-standard cumulative deficit and phenotypic measures, and have been shown to predict outcomes including hospital readmission and mortality (17, 18). In post hoc analyses, we did not find that any single, heavily weighted diagnosis was driving higher HFRS scores in either breast cancer survivors or comparison subjects; instead we observed that several unspecified types of conditions (e.g., other soft tissue disorder, not elsewhere classified) were most common. This may suggest that frailty in this population is defined as a vulnerable state, observed by the accumulation of multiple conditions.

The present study was conducted among women breast cancer survivors, who may be at increased risk of frailty due to several factors. Evidence from childhood cancer survivors suggests that female survivors are at greater risk of developing frailty than male cancer survivors (27, 28), perhaps because women have a lower ability to regenerate muscles (29), and women whose treatment affects estrogen production (e.g., breast cancer patients) are even more prone to frailty (30). Cancer survivors have also been shown to be at higher risk of other geriatric conditions, even at younger ages. Indeed, it was shown that breast cancer survivors have higher risk of developing cardiovascular disease, and, as in this study, the risk difference was greatest among those diagnosed at younger ages (23). The evidence from the present study expands on these earlier findings by exploring breast cancer diagnosis in a much larger sample across a greater range of ages and using ≥20 years of follow-up.

Although some of these negative effects may be caused by the cancer itself, it seems that treatment does most of the long-term damage (28, 31), mimicking the effects of aging (32). Cancer treatments and the aging process share one important feature—the accumulation of damage over time (33). DNA mutations are shared by cancer patients and aging individuals, meaning that aging individuals are at substantially increased risk for cancer (34), but this shared mechanism suggests a bidirectional relationship, meaning that cancer patients/survivors are at risk of more rapid aging, too (7, 35). Recent findings have suggested that chemotherapeutics may potentially cause gerontogenicity—that is, have the ability to accelerate aging (31). Our finding that the association between breast cancer survivorship and frailty was weaker when survivors of other forms of cancer were included in the comparison group supports this point, because including survivors of other forms of cancer in the comparison group would mask this association. This hypothesis is supported in other studies, as well. Among men, prostate cancer is the most common form of cancer and is often treated with androgen deprivation therapy. Studies have shown that androgen deprivation therapy is associated with frailty, osteoporosis, muscle wasting, and weakness in survivors, and users had a prevalence of frailty of approximately 40%, while only 15% of nonusers were frail (36).

Importantly, inflammation is a link between cancer and aging. Inflammation generally increases even in healthy aging (37, 38), and chemotherapy agents have aging-like proinflammatory effects (39). Preclinical models have shown that radiation and genotoxic and cytotoxic cancer therapeutics cause physiological changes that mirror the molecular and cellular hallmarks of the aging process (33)—most relevantly, increased inflammation (40, 41). The limited evidence from clinical samples found that a higher concentration of C-reactive protein, a proinflammatory marker, was associated with mortality risk among cancer survivors (42). Aging research has focused a great deal on understanding the role of inflammation in these processes and risks, but little information is available on the role of inflammation in the association between cancer survivorship and risk of frailty. It is critical to understand, though, because it may be a predictive and/or mediating factor. Other aging-related mechanisms, including oxidative stress, disrupted protein homeostasis, and epigenetic alterations, may also contribute to the gerontogenicity observed in cancer survivors (26).

Another possible explanation for the functional decline observed in cancer survivors over the long term may instead relate to long-term chemotherapy-induced peripheral neuropathy, which is present in 40% of breast cancer survivors 2 years after treatment initiation (43). Peripheral neuropathy has been associated with poorer functional ability, balance deficits, and falls (44). Additionally, other factors, including lifestyle changes or diet, may further contribute to the development of aging-related conditions among cancer survivors. During treatment, cancer patients show evidence of sarcopenia, compounded or driven by poor nutritional intake, oxidative stress, hormonal disruption, and decreased physical activity (45). These factors may directly or indirectly affect risk of frailty in the short and long term, and we cannot rule out confounding effects from them. Future studies investigating these potential pathways will be important. However, understanding the risk and patterns of frailty is the first step to understanding the scope of the problem, prior to understanding the mechanisms.

This study has multiple strengths, including the large population-based sample, the over 20 years of follow-up, and the lack of attrition, although the findings must also be considered within the study’s limitations. First, inclusion in this study hinged on surviving cancer, because the goal of the study was to examine frailty among breast cancer survivors. Survivorship was defined as living 5 years after the initial diagnosis, which is standard in clinical practice. However, these survivors may be healthier than those who die prior to the 5-year timepoint, thus introducing a survivor bias to the “true” effect of frailty risk from cancer or cancer treatment. We examined frailty prior to diagnosis and during the 5-year period between diagnosis and the start of follow-up for frailty, to ascertain the differences between survivors and comparisons, to the extent possible. We found that, at all diagnosis ages, the mean frailty scores prior to diagnosis and start of follow-up were similar in breast cancer survivors and comparisons. We additionally examined risk of frailty including the first 5 years from diagnosis, and found the association attenuated towards the null. This period is murkier in terms of survivorship, as many of the women diagnosed with cancer died in this period and, clinically, they were not as likely to be “cancer-free.” Further, frailty typically does not have an acute onset; it may take more time to develop in survivors. Therefore, the specific aim of this study was to examine the long-term outcomes in survivors who had lived for 5 years beyond their diagnosis. Second, past studies have shown that among breast cancer survivors, there are racial differences in risk of developing cardiovascular disease (23). Because this study used Swedish registry data, most participants were Swedish, and data on race/ethnicity was not available. We adjusted for country/region of birth, but this is only a proxy for race/ethnicity; therefore, our findings may not be directly generalizable to more diverse populations. Third, the HFRS was designed as a screening measure for frailty in clinical populations, and although it shows overlap with gold-standard frailty measurement tools (17), it may not be sensitive enough to capture the “healthier frail” individuals, particularly those who did not seek medical care and would not have been captured in the register. Although this method allowed us to measure frailty in the national registers, this approach likely led to an underestimation of frailty in both survivors and comparisons—potentially to a greater degree among comparisons as they may be less likely to seek clinical care. Finally, because we hypothesized that cancer treatment is the driver of gerontogenicity, it may have been useful to account for specific types of treatment. The aim of this study was to be more descriptive and map out the incidence of frailty in cancer survivors, rather than looking specifically at any individual treatments. Future studies should consider adjusting or stratifying for type of treatment, to better understand the differences associated with frailty and among other types of cancer.

As cancer survivorship increases and survivors cope with aging-related conditions (4), understanding the health risks for this population is critical, because studies have shown that quality of life is of utmost importance to them (14). The findings from this study suggest that risk of frailty has increased in more recent years, so this may be gradually becoming a more pressing clinical concern. Frailty among cancer survivors has been associated with multiple adverse outcomes, including hospitalization, chronic disease onset, and mortality (26). Importantly, frailty can be intervened upon using pharmaceutical, nutraceutical, or lifestyle interventions. Indeed, studies among breast cancer survivors have shown that restorative exercise programs implemented among cancer patients and survivors are beneficial for working towards preventing these outcomes (46). This suggests that frailty can be ameliorated, reversed, or even avoided, but it is likely that the earlier the intervention, the more effective it will be. Efforts to support aging cancer survivors must be multidisciplinary. From a clinical standpoint, the management will go beyond oncologists and geriatricians, and general practitioners must be involved in the development of care guidelines and plans (5). However, the first step must be to define and understand the risk of frailty and related outcomes in cancer survivors, to understand when is best to intervene to promote healthy aging in this growing population.

## Supplementary Material

Web_Material_kwad048
